# Aquaporin-4 (AQP4) antibody-positive neuromyelitis optica spectrum disorder (NMOSD) complicated with acute pancreatitis: A case report

**DOI:** 10.1097/MD.0000000000047833

**Published:** 2026-02-28

**Authors:** Siwei Luo, Yang Yang, Ting Long, Xiaoyan Guo

**Affiliations:** a Department of Neurology, The Affiliated Hospital of Southwest Medical University, Luzhou, Sichuan, China.

**Keywords:** acute pancreatitis, AQP4, aquaporin-4, neuromyelitis optica spectrum disorder, NMOSD

## Abstract

**Rationale:**

Neuromyelitis optica spectrum disorders (NMOSD) is an autoimmune-mediated central nervous system (CNS) inflammatory demyelinating disease characterized by 6 major clinical syndromes, including myelitis, optic neuritis, area postrema syndrome, symptomatic cerebral syndrome, brainstem syndromes, and acute diencephalic clinical syndrome.

**Patient concerns:**

A 32-year-old female, complaining of numbness in her right limb for 1 month, was diagnosed with aquaporin-4 antibody (AQP4-IgG)-positive NMOSD. She developed acute pancreatitis (AP) during disease relapse, representing a rare extra-neurological manifestation of NMOSD.

**Diagnoses:**

Spinal contrast-enhanced magnetic resonance imaging scan indicated hyperintense lesions on the T2-weighted sequence in the spinal cord at C2/C4 at the initial onset and extending from C2 to C6 vertebral levels at the recurrence of the disease. Serological profiling demonstrated a significant rise in AQP4-IgG titers (from 1:100 to 1:1000). During the recurrence of NMOSD, the patient manifested acute gastrointestinal symptoms, including severe epigastric pain with emesis. Laboratory tests revealed profound hyperamylasemia (amylase, 2293.8 U/L; pancreatic amylase, 1843.6 U/L). Abdominal computed tomography scan demonstrated definitive signs of AP, confirming the diagnosis.

**Interventions:**

She received methylprednisolone and mycophenolate mofetil for NMOSD treatment. When AP occurred, she was placed on nil per os status and treated with gabexate and esomeprazole.

**Outcomes:**

The patient was discharged with relief of neurological dysfunction symptoms and maintained on nasojejunal tube feeding for nutritional support. Three months after discharge, clinical assessment revealed stable myelitis symptoms. There was no evidence of pancreatitis recurrence during the follow-up period.

**Lessons:**

This case suggests that AQP4-IgG-mediated immune damage may not be confined to the CNS. There may be a possible association between NMOSD and AP in the pathophysiological mechanisms. AP may be a rare extra-CNS complication of NMOSD. Our case expands the spectrum of potential systemic complications in NMOSD, highlighting the need for increased clinical vigilance.

## 1. Introduction

Neuromyelitis optica spectrum disorders (NMOSD) is an autoimmune-mediated central nervous system (CNS) inflammatory demyelinating disease characterized by 6 major clinical syndromes, including myelitis, optic neuritis, area postrema syndrome (APS), symptomatic cerebral syndrome, brainstem syndromes, and acute diencephalic clinical syndrome.^[1]^ The pathogenesis is predominantly associated with aquaporin-4 antibody (AQP4-IgG).^[1]^ Herein, we report an AQP4-IgG-positive NMOSD patient who developed acute pancreatitis (AP) during relapse and further discuss whether underlying pathophysiological mechanisms may explain the co-occurrence of NMOSD and AP. To our knowledge, this possible association between NMOSD and AP has not been reported or discussed before.

## 2. Case presentation

**Figure 1.**
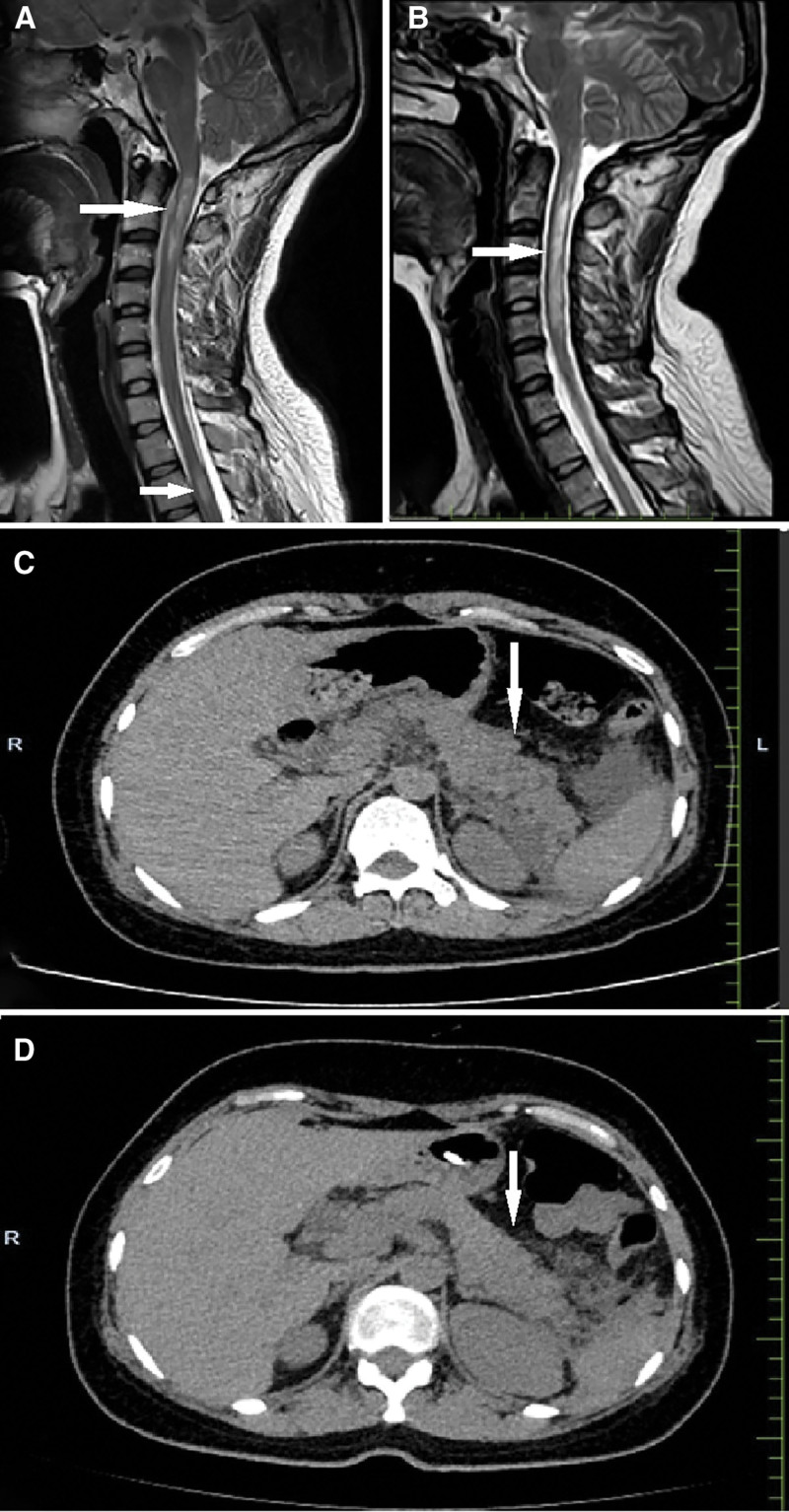
Spinal MRI scans on the T2-weighted sequence showed 2 hyperintense lesions at C2/C4 and T2 vertebral levels (arrow) at the initial onset (A) and extending from C2 to C6 vertebral levels (arrow) at the recurrence of the disease (B). Abdominal CT scan showed typical signs of AP (arrow), including enlargement of the pancreatic body and tail with irregular contours, blurred surrounding fat spaces, and fluid accumulation (C), and significant improvement in pancreatitis imaging findings after treatment (D). AP = acute pancreatitis, CT = computed tomography, MRI = magnetic resonance imaging.

**Figure 2.**
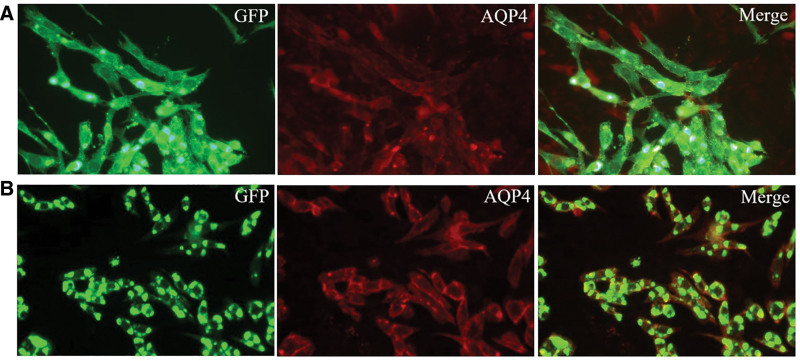
Immunofluorescence of anti-AQP4 antibodies in the patient’s serum. Fluorescent antibody staining for expression of AQP4 antibody at the initial onset (A) and recurrence (B). AQP4 = aquaporin-4.

A 32-year-old Chinese Han female was admitted to the neurology department of our hospital complaining of numbness in her right limb for 1 month, with no other neurological dysfunction symptoms and no family history of neurological disease. A neurological examination was unremarkable except for hypoalgesia affecting the right hemibody. Her brain magnetic resonance imaging (MRI) showed no pathological findings. A subsequent spinal contrast-enhanced MRI scan indicated 2 hyperintense lesions on the T2-weighted sequence in the spinal cord at C2/C4 and T2 vertebral levels (Fig. 1A), suggestive of a possible case of myelitis. The cerebrospinal fluid (CSF) analysis demonstrated normal opening pressure (120 mm H_2_O), mild pleocytosis (white blood cell count 25 × 10^6^/L, 80% mononuclear cells), slightly elevated protein level (0.423 g/L), slightly lower glucose concentration (2.79 mmol/L), normal chloride and lactate levels, and negative results for Gram staining, smear staining, and bacterial and fungal smears. Meanwhile, CSF and serum detection for antibodies (AQP4, myelin oligodendrocyte glycoprotein, myelin basic protein, glial fibrillary acidic protein) for immunopathological evaluation were conducted on a cell-based assay. AQP4-IgG were positive in the serum (1:100) but negative in the CSF (Fig. 2A). The testing for antibodies (myelin oligodendrocyte glycoprotein, myelin basic protein, glial fibrillary acidic protein) was negative in the serum and CSF. Oligoclonal bands were found to be negative. She was diagnosed with AQP4-IgG-positive NMOSD and received intravenous methylprednisolone therapy in a tapering regimen: 1000 mg/day for 5 days, followed by 500 mg/day for 3 days, 250 mg/day for 3 days, and finally 120 mg/day for 3 days. She was discharged with improved paresthesia and treated with oral prednisolone acetate (60 mg/day, tapered by 10 mg weekly to a maintenance dose of 10 mg/day) and mycophenolate mofetil (1500 mg/day). At 22 months after the initial episode, the patient was readmitted with recurrent right upper limb numbness and weakness. Spinal MRI demonstrated progressive cervical cord lesions extending from C2 to C6 vertebral levels (Fig. 1B) and no detectable thoracic cord lesions, compared with the previous episode. Serological profiling demonstrated a significant rise in AQP4-IgG titers (from 1:100 to 1:1000; Fig. 2B). A diagnosis of NMOSD relapse was made, and she was treated with intravenous methylprednisolone for 1000 mg/day. By the fourth day of immunotherapy, the patient manifested acute gastrointestinal symptoms, including severe epigastric pain with emesis. Abdominal assessment showed diffuse distension and localized tenderness in the right hypochondriac region. Abdominal ultrasound demonstrated abundant gallstones in her gallbladder. Laboratory tests revealed profound hyperamylasemia (amylase, 2293.8 U/L; pancreatic amylase, 1843.6 U/L). Abdominal computed tomography scan demonstrated definitive signs of AP (Fig. 1C), confirming the diagnosis. The patient was placed on nil per os status and treated with intravenous gabexate (300 mg/day) and esomeprazole (80 mg/day). Given the rising inflammatory markers, intravenous cefoperazone-sulbactam (3 g q8h/day) was initiated while methylprednisolone and mycophenolate mofetil were temporarily withheld. After 2 weeks of treatment, the patient achieved complete resolution of abdominal pain with normalization of serum amylase and pancreatic amylase levels. Follow-up abdominal computed tomography demonstrated significant improvement in pancreatitis imaging findings (Fig. 1D). Then, she was discharged and maintained on nasojejunal tube feeding for nutritional support. Oral prednisolone acetate (50 mg/day) was reintroduced at 1 month after discharge, followed by incorporation of mycophenolate mofetil (1500 mg/day) at 2 months as maintenance immunotherapy for NMOSD recurrence prevention. Three months after discharge, clinical assessment revealed stable myelitis symptoms. There was no evidence of pancreatitis recurrence during this follow-up period.

## 3. Discussion

Aquaporins (AQPs) are a family of transmembrane proteins that facilitate the transport of water, glycerol, ammonia, urea, H_2_O_2_, and other small molecules across biological membranes.^[2]^ Within the CNS, AQP4 localizes primarily to astrocytes, especially highly enriched in the blood-brain barrier, optic nerve, and periventricular regions (such as the postrema, hypothalamus, and brainstem), and is involved in regulating water homeostasis and neural excitability.^[3]^ The primary pathogenesis of NMOSD involves AQP4-IgG binding to AQP4 on astrocytes, initiating complement activation, inflammatory cell recruitment, astrocytic injury, and secondary inflammatory cascades culminating in demyelination, neuronal injury, and release of pro-inflammatory mediators.^[1]^ Relapse course is very common in the clinical course of NMOSD, with approximately 60% of patients experiencing a relapse within the first year and 90% within 3 years.^[4]^ This case experienced a myelitis relapse with coincident AP with markedly elevated AQP4-IgG titer (1:1000 vs baseline 1:100). The potential association between AP onset and elevated AQP4-IgG titers remains undetermined. Notably, AQPs are functionally expressed in both endocrine and exocrine pancreatic compartments, where they critically regulate fundamental physiological processes in insulin secretion and pancreatic fluid secretion.^[5]^ In humans, although several AQPs mRNA (AQP1, AQP3, AQP4, AQP5, AQP8, AQP12) have been detected in the exocrine pancreas, the protein expressions have been confirmed only for AQP1, AQP5, and AQP8.^[6]^ While AQP4 mRNA is detectable, its protein localization and physiological role in pancreatic cellular function remain to be elucidated.

The predominant etiology of AP is cholelithiasis, followed by hypertriglyceridemia and excessive alcohol consumption.^[7]^ Other causes include drugs, endoscopic retrograde cholangiopancreatography, hypercalcemia, infection, genetic predisposition, autoimmune disorders, and trauma.^[8]^ Our reported patient presented with normal lipid profiles, no history of alcohol consumption, and other risk factors. The identification of abundant gallstones within her gallbladder strongly suggests biliary lithiasis as the direct etiology of AP. However, before the initial diagnosis of NMOSD, her medical records showed that the abdominal ultrasound did not reveal any gallstones in her gallbladder. Notably, at the level of the liver, bile ducts, and gallbladder, AQPs play a critical pathophysiological role in pathways of hepatobiliary secretion and are associated with several hepatobiliary disorders, including cholestatic diseases and cholesterol cholelithiasis.^[9]^ AQP4 mediates the composition regulation of bile components through the mechanism of water reabsorption/secretion in the biliary tract.^[10]^ AQP4 is localized to the basolateral cholangiocyte membrane, which exhibits close anatomical proximity to the peribiliary vascular plexus.^[11]^ Peribiliary vascular plexus surrounding the bile ducts is related to the water secretion of the biliary system. Experimental studies have demonstrated that AQP4 facilitates water transport across cholangiocyte membranes, thereby playing a critical role in bile formation.^[10,11]^ Therefore, in this case, we postulate that the AQP4-IgG may contribute to the pathophysiological mechanisms of gallstone formation. This antibody-mediated process could represent a novel pathogenic pathway for AP in NMOSD. The precise role of AQP4 in the physiological mechanism of bile formation and secretion remains not completely clear and requires further clinical and basic research studies.

AQP4 is highly expressed in the area postrema (chemoreceptor trigger zone for emesis) in the CNS. This anatomical specificity underlies why APS, characterized by intractable nausea, vomiting, and hiccups, represents a pathognomonic clinical phenotype of NMOSD.^[12]^ Therefore, the patients of APS were usually initially admitted to the gastroenterology department and misdiagnosed as a gastrointestinal disease with the subsequent diagnostic delay. Notably, 1 year prior to the onset of initial myelitis symptoms, the patient had experienced recurrent episodes of vomiting and nausea, for which she visited the gastroenterology clinic multiple times. Initial evaluations, including abdominal ultrasonography, revealed no structural abnormalities, and her symptoms showed intermittent improvement with symptomatic treatment. It was only after the onset of limb paresthesia that she was definitively diagnosed with NMOSD. Her pre-myelitis gastrointestinal symptoms probably reflect undiagnosed APS and not necessarily early pancreatic involvement. During disease relapse, the NMOSD patient exhibited a significant elevation in AQP4-IgG titer, was found to have abundant gallstones, and subsequently developed AP. This case suggests a possible extra-CNS pathogenic role of AQP4-IgG. With the further understanding of the immunobiology of AQP4 autoimmunity, researchers have proposed replacing the term “NMOSD” with “autoimmune AQP4 channelopathy” when referring to AQP4 autoimmunity.^[13]^

As a single case report, our findings lack generalizability. The evidence provided is inherently descriptive and cannot establish a definitive causal relationship between the conditions. Despite these limitations, this report offers valuable insights into possible extra-CNS complications of NMOSD and highlights an area worthy of further research.

## 4. Conclusion

Early gastrointestinal symptoms should alert clinicians to potential CNS disorders. Our case enhances the understanding of digestive system complications and expands the spectrum of potential systemic complications in NMOSD, highlighting the need for increased clinical vigilance. This case suggests a possible association between NMOSD and AP. AQP4-IgG-mediated immune damage may not be confined to the CNS. AP may represent a rare extra-CNS complication of NMOSD. The onset of complications can complicate NMOSD treatment. Potential molecular mechanisms may underlie the association between NMOSD and AP, and require further research to elucidate.

### 4.1. Patient perspective

When I had vomiting and nausea at onset, I thought that I just suffered from gastrointestinal disease. It was only after the onset of limb paresthesia that I was definitively diagnosed with NMOSD. However, when the disease relapsed with AP concurrence, I felt very frightened. As therapy went on, my symptoms gradually stabilized and there has been no recurrence since then. But I’m still a little worried about relapse of the disease.

## Author contributions

**Data curation:** Siwei Luo, Yang Yang, Ting Long.

**Formal analysis:** Siwei Luo, Yang Yang, Ting Long.

**Writing – original draft:** Siwei Luo, Xiaoyan Guo.

**Writing – review & editing:** Siwei Luo, Xiaoyan Guo.

## References

[1] Siriratnam P Huda S Butzkueven H van der Walt A Jokubaitis V Monif M. A comprehensive review of the advances in neuromyelitis optica spectrum disorder. Autoimmun Rev. 2023;22:103465. 37852514 10.1016/j.autrev.2023.103465

[2] Galli M Hameed A Żbikowski A Zabielski P. Aquaporins in insulin resistance and diabetes: more than channels! Redox Biol. 2021;44:102027. 34090243 10.1016/j.redox.2021.102027 PMC8182305

[3] Prasad S Chen J. What you need to know about AQP4, MOG, and NMOSD. Semin Neurol. 2019;39:718–31. 31847043 10.1055/s-0039-3399505

[4] Ma X Kermode AG Hu X Qiu W. NMOSD acute attack: understanding, treatment and innovative treatment prospect. J Neuroimmunol. 2020;348:577387. 32987231 10.1016/j.jneuroim.2020.577387

[5] Arsenijevic T Perret J Van Laethem JL Delporte C. Aquaporins involvement in pancreas physiology and in pancreatic diseases. Int J Mol Sci. 2019;20:5052. 31614661 10.3390/ijms20205052 PMC6834120

[6] Calamita G Delporte C. Insights into the function of aquaporins in gastrointestinal fluid absorption and secretion in health and disease. Cells. 2023;12:2170. 37681902 10.3390/cells12172170 PMC10486417

[7] Gardner TB. Acute pancreatitis. Ann Intern Med. 2021;174:ITC17–32. 33556276 10.7326/AITC202102160

[8] Boxhoorn L Voermans RP Bouwense SA. Acute pancreatitis. Lancet. 2020;396:726–34. 32891214 10.1016/S0140-6736(20)31310-6

[9] Khalil M Gena P Di Ciaula A Portincasa P Calamita G. Aquaporins in biliary function: pathophysiological implications and therapeutic targeting. Int J Mol Sci. 2024;25:12133. 39596202 10.3390/ijms252212133 PMC11593884

[10] Splinter PL Masyuk AI Marinelli RA LaRusso NF. AQP4 transfected into mouse cholangiocytes promotes water transport in biliary epithelia. Hepatology. 2004;39:109–16. 14752829 10.1002/hep.20033

[11] Marinelli RA Pham LD Tietz PS LaRusso NF. Expression of aquaporin-4 water channels in rat cholangiocytes. Hepatology. 2000;31:1313–7. 10827157 10.1053/jhep.2000.7986

[12] Dubey D Pittock SJ Krecke KN Flanagan EP. Association of extension of cervical cord lesion and area postrema syndrome with neuromyelitis optica spectrum disorder. JAMA Neurol. 2017;74:359–61. 28097302 10.1001/jamaneurol.2016.5441

[13] Hinson SR Lennon VA Pittock SJ. Autoimmune AQP4 channelopathies and neuromyelitis optica spectrum disorders. Handb Clin Neurol. 2016;133:377–403. 27112688 10.1016/B978-0-444-63432-0.00021-9

